# Case Report: MR-guided adaptive stereotactic body radiotherapy for splenic metastases: a case series of technical feasibility and early clinical outcomes

**DOI:** 10.3389/fonc.2026.1905280

**Published:** 2026-09-15

**Authors:** D. Tan, L. Edwards, H. L. Byrne, T. Liang, K. Biggerstaff, E. Leong, T. Lim, K. Rahim, H. Tan

**Affiliations:** 1 Genesis Care Murdoch, Murdoch, WA, Australia; 2 Faculty of Medicine and Health, The University of Sydney, Camperdown, NSW, Australia; 3 Department of Surgery, UWA Medical School, The University of Western Australia, Crawley, WA, Australia

**Keywords:** MRI radiation therapy, oligometastases, SBRT (stereotactic body radiation therapy), spleen, stereotacic radiation

## Abstract

We present a case series of four patients with five splenic metastases treated with stereotactic body radiation therapy (SBRT) using a 1.5-T Unity (Elekta AB, Stockholm, Sweden) magnetic resonance linear accelerator (MR-Linac). While SBRT is a standard of care for the treatment of oligometastatic disease, splenic SBRT is not well established due to its rarity. MRI-guided adaptive radiotherapy (MRgART) offers superior soft tissue visualization and the unique ability to adapt treatment plans in real time. In our series, splenic oligometastases originated from primary malignancies of melanoma, colorectal adenocarcinoma, and non-small-cell lung cancer. Prescription doses ranged from 40 to 50 Gy in five fractions, with gross tumor volumes between 2.4 and 22.7 cm^3^. At 12 months, the local control was 80%. One patient developed grade 2 lymphopenia without infection following two courses SBRT with a total mean spleen dose of 24.4 Gy. Our experience suggests that MRgART is a technically feasible, effective, and non-invasive treatment for splenic metastases, offering excellent disease control with an acceptable toxicity profile. However, the benefits of splenic SBRT should be balanced against the potential risk of hematological toxicity, and prophylactic vaccinations for hyposplenism should be considered for this patient population.

## Introduction

This case series presents four cases of splenic metastasis treated with magnetic-resonance-guided adaptive radiation therapy (MRgART) on a 1.5-T Unity (Elekta AB, Stockholm, Sweden) magnetic resonance linear accelerator (MR-Linac).

While splenic metastases are common, isolated splenic oligometastasis is rare, occurring in less than 1% of oligometastatic cancer cases. These lesions are generally asymptomatic (1, 2), and commonly arise from colorectal, ovarian, breast, lung, and melanoma primaries. They may present in either a synchronous or metachronous fashion. Imaging features include a hypoechoic/hypoattenuating splenic mass on US or CT, respectively, while the MRI and PET findings generally mimic the primary malignancy (1). Differential diagnoses include lymphoma, hemangioma, fungal disease, septic emboli, and granulomatous disease; however, tissue diagnosis via US- or CT-guided biopsy carries a significant risk of major bleeding (1, 3).

Management of splenic oligometastases is heterogeneous due to its rarity. Surgical splenectomy may be considered, but it is a major surgery with significant surgical morbidity and lifelong immunosuppression (2).

A national quality improvement program study reported on 1,344 patients who underwent splenectomy. Those with malignancy had a 30-day major complication rate of 27%, mean hospital length of stay of 7.7 days, and 1.6% mortality rate compared to a 14% major complication rate for splenectomy for benign conditions (4). Major complications include reoperation, bleeding, sepsis, intensive care/organ replacement therapy, and thromboembolic events.

Recent retrospective single-center studies of splenectomy for malignancy report heterogeneous outcomes, ranging from laparotomy 7%–86%, mean length of hospital stay between 3 and 14 days, major complications ranging 13%–48%, and 30-day mortality of 2% to 3% (5, 6).

A laparoscopic approach may be suitable for patients without splenomegaly, and a metanalysis of published case series found less morbidity compared to open surgery (OR 0.5), 2.5 day shorter length of hospital stay, and less mortality (OR 0.4), which may account for some of the wide range in outcomes reported above (7).

Stereotactic body radiotherapy (SBRT) is now commonly used to treat oligometastatic disease in many organs, including liver, lung, adrenal, and lymph nodes. Its application to spleen remains limited due to challenges regarding target motion, proximity to gastrointestinal organs, and uncertainty regarding the effects of splenic irradiation on immune function and lymphopenia.

To our knowledge, there are only five published case reports of splenic SBRT, and all were treated on either a conventional or a robotic Linac platform (with fiducial guidance) (8–12). The stomach, small intestine, and the splenic flexure of the colon are often adjacent to the spleen and are considered critical dose-limiting organs at risk (OAR). These intraperitoneal structures exhibit significant inter- and intrafraction motion due to respiration, peristalsis, and variations in luminal contents (13). MRgART enables real-time organ motion management, adaptive planning, and superior soft tissue delineation, allowing precise and safe SBRT delivery without the requirement for invasive fiducial marker placement.

We report our initial single-institutional experience of the safety and efficacy of MRgART treatment of splenic metastases in four patients (five lesions).

## Case presentations

Patients were treated with MRgART on a Unity MR-Linac to a single oligometastatic splenic metastasis between June 2023 and January 2025. Data were collected under the ADAPT registry (2020/ETHO1414) (14) and GenesisCare Outcomes Protocol (2022/ETHO0247), both approved by the St. Vincent’s Darlinghurst, Sydney Human Research Ethics Committee, and written informed consent was obtained from all patients. All patients were discussed at a multidisciplinary tumor board and peer-reviewed prior to treatment. Indications for SBRT were either a new solitary splenic metastasis (metachronous) or oligoprogression of a splenic metastasis. Splenic metastases were diagnosed based on characteristic imaging features on FDG-PET and CT imaging. Biopsy was not performed due to procedural risk. The patients’ characteristics are summarized in **Table 1**.

**Table 1:** Patient and treatment characteristics.

Patient	A	B	C	D-1	D-2
Primary tumour	Colorectal	Lung NSCLC EGFRm	Melanoma	Melanoma	Melanoma
Age	80s	50s	40s	50s	--
Axial dimension (mm)	13	9	15	26	24
Prescription dose (Gy), all in five fractions	50	40	45	40	40
GTV D100%	53.4	41.5	48.4	44.1	30
Mean spleen dose (Gy)	22.5	16.6	12.3	14.8	9.6
Outcome	CMR 14 months	CMR 26 months	Pseudo/progression 2 months; CMR 18 months	CMR 14 months	CMR 12 months
Toxicity	Nil	Nil	Nil	G1 fatigue	G1 fatigue, G2 lymphopenia

Case A was a female patient in her 80s who was initially diagnosed with T3N1M1 adenocarcinoma of the sigmoid colon, with one liver and one lung metastasis. She received chemotherapy (FOLFOX and then XELIRI) with complete metabolic response in the primary and nodes but had residual lung and liver metastasis. Both were treated with stereotactic radiotherapy (50 Gy in five fractions to segment 5 liver and right middle lobe lung). At 2 years from diagnosis, she had progression in the sigmoid colon, which was treated surgically with an anterior resection. At 3 years from diagnosis, a metachronous splenic metastasis was found on FDG-PET, consisting of an avid 13-mm nodule in the medial subcapsular spleen. She declined surgery and proceeded to splenic SBRT (50 Gy/5 fractions) with MRgART.

After SBRT, the lymphocyte counts were stable. After 2 months, the maximum standardized uptake value (SUVmax) fell from 4.7 to 3.9, and after 5 months she had a complete metabolic response. Unfortunately, she developed a new liver metastasis and had further treatment with capecitabine chemotherapy and liver SBRT with complete response. Subsequently, she had peritoneal metastases and passed away after 14 months.

Case B was a female patient in her 50s who was diagnosed with T2N1M1 non-small-cell lung cancer (adenocarcinoma with exon 19 EGFR mutation) with metastatic disease to multiple bones. She responded well to systemic treatment (afatinib) with complete metabolic response in all metastases, and the residual primary was treated surgically (left lower lobe VATS lobectomy). At 5 years after diagnosis, she had a pathologically confirmed recurrence in the mediastinal node, and her treatment was changed to osimertinib with complete response. At 6 months later, a new splenic metastasis was found on FDG-PET with an avid 9-mm lesion in the anterior spleen. She proceeded to splenic SBRT (40 Gy/5 fractions) with MRgART, with the stomach abutting and limiting dose. After SBRT, the lymphocyte counts were stable. At 9 months after SBRT, she had a complete response on FDG-PET, and at 26 months she has had no evidence of the disease (she remains on osimertinib).

Case C was a male patient in his 40s who was diagnosed with T2N0M0 nodular melanoma of the left leg. This was excised, and no adjuvant therapy was given. At 2 years later, he developed metastases to the bones, brain, liver, and spleen and commenced on nivolumab. After 1 year, he developed oligoprogression in multiple left external iliac nodes and was treated with hypofractionated VMAT radiotherapy (50 Gy/20 fractions). At 3 months later, he had further oligoprogression in the lateral spleen, with a 15-mm FDG-avid lesion. He proceeded to splenic SBRT (45 Gy/5 fractions) with MRgART.

At 2 months after spleen SBRT, the lesion enlarged to 24 mm and increased the SUVmax from 4.7 to 14. He also had new nodal disease (common iliac) and infield progression of external iliac nodes. His treatment was changed to encorafenib and binitinib, with ongoing complete metabolic response at 18 months. Due to the change in systemic therapy, it was not possible to determine whether the spleen changes were pseudoprogression or true progression.

Case D was a male patient in his 50s who presented with metastatic melanoma of the left forearm, with multiple metastases in his lungs, spleen, adrenal, liver, and bones. He was started on nivolumab and ipilimumab, followed by maintenance nivolumab with a very good partial response. After 2 years of immunotherapy, he had oligopersistent lumbar spinal disease, treated with SBRT (27 Gy/3 fractions) with complete metabolic response, and his immunotherapy was stopped. At 4 years after diagnosis, he developed a solitary recurrence, with a 26-mm FDG-avid lesion in the splenic hilum. He was treated with spleen SBRT (40 Gy/5 fractions) with MRgART, with a single dose of nivolumab before and after.

At 3 months after spleen SBRT, the FDG-PET showed a partial response with SUVmax 3.4 reduced from 4.5, but it also showed a new 24-mm splenic metastasis anteroinferior to the prior lesion. This new lesion was treated with spleen SBRT (40 Gy/5 fractions) with MRgART. The internal target volume (ITV) abutted the prior SBRT volume, with 2cm^3^ deep within the spleen receiving a more than 80 Gy EQD2. The small bowel was in close proximity anteriorly and inferiorly so that the border of the gross tumor volume (GTV) received an isotoxic dose (~30–41 Gy). After 2 months, he developed grade 2 lymphopenia (0.6 ×10^9^/L), which resolved after 4 months. At 12 months after the second spleen MRgART SBRT, he had complete metabolic response in both lesions. He had no acute or late gastrointestinal toxicity.

## Radiation treatment planning details

MR-guided adaptive SBRT with a dose of 40–50 Gy in five fractions was prescribed to be delivered on non-consecutive days. Given the rarity of splenic SBRT, dose selection was extrapolated from established adrenal and liver SBRT regimes. Each case was prospectively discussed and the treatment plan reviewed at the internal MR-Linac peer review meeting (15).

Simulation was performed with abdominal compression to reduce respiratory motion. The patients were positioned supine with their arms by their sides, away from the body, for comfort. Respiratory motion was assessed using 4DCT and BTFE MRI. An ITV was created based on the motion, and then a uniform 5-mm planning target volume (PTV) expansion was applied.

Treatment planning was performed using the Monaco treatment planning system (TPS; Elekta, Stockholm, Sweden). A reference plan was created using eight to nine IMRT 7MV beams and underwent patient-specific quality assurance. The stomach, duodenum, large bowel, and/or small bowel structures served as the primary dose-limiting structures for the majority (80%) of cases. Axial and sagittal MRI with radiation plan and OAR can be seen in **Figure 1**.

**Figure 1:**
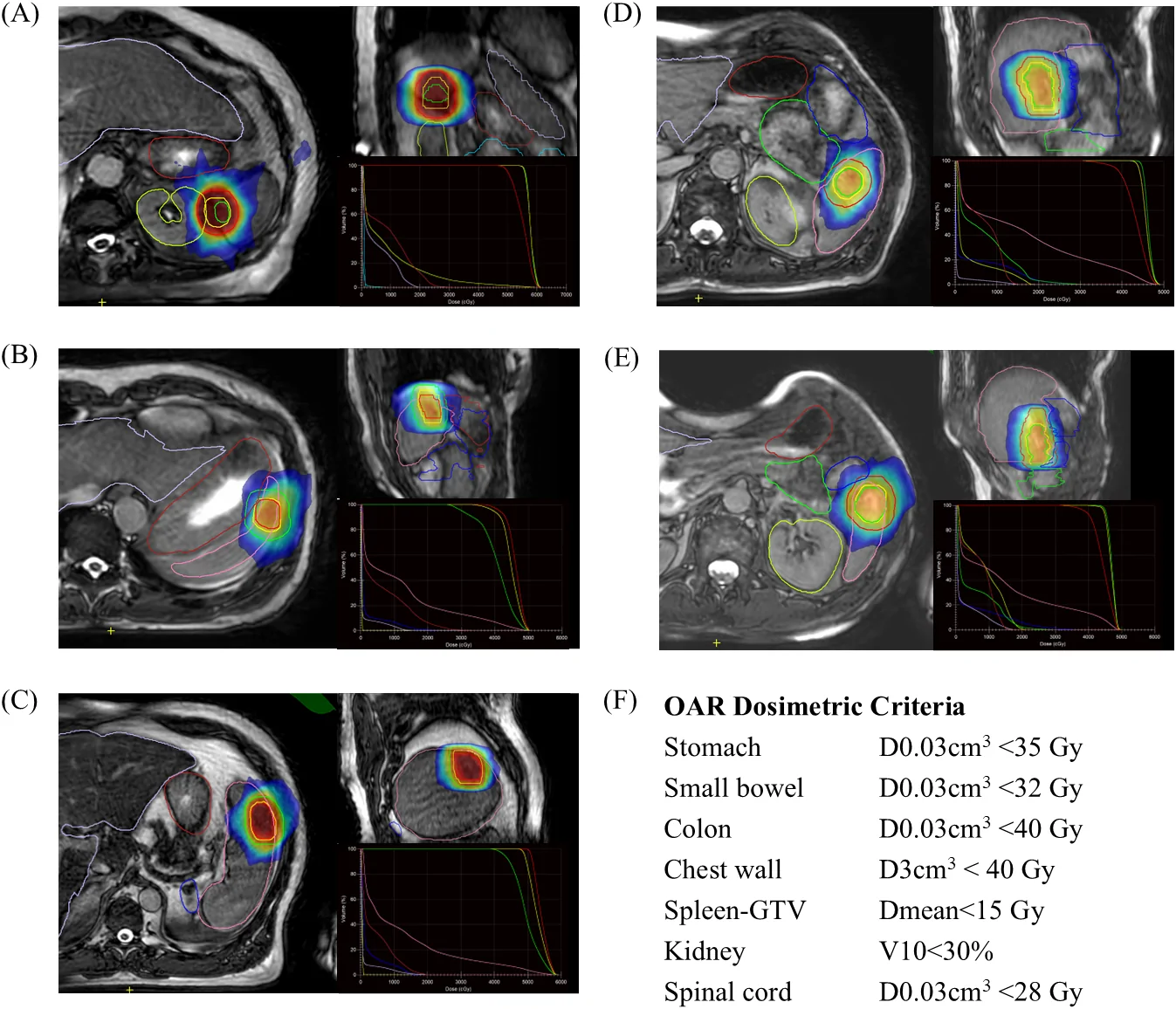
**(A–E)** Axial and sagittal MRI at fraction 1 for patients **(A–D**-1**)**, and D-2, respectively. **(F)** OAR dosimetric criteria. Target volumes: GTV, green; ITV, yellow; PTV, red. OAR contours: stomach, brown; colon, blue; small bowel, dark green; kidney, yellow. Color-wash radiation dose: blue, 23 Gy; cyan, 35 Gy; yellow, 45 Gy; orange, 50 Gy; dark red, 55 Gy.

## Treatment details

Treatment was delivered on the Unity MR-Linac (Elekta, Stockholm, Sweden) over 2.5 weeks at a frequency of two fractions per week. For each fraction, the patients fasted for 3 h prior to treatment and received pre-treatment medications 30 min before radiotherapy (buscopan—20 mg and ondansetron—4 mg). An adapt-to-shape (ATS), dual-imaging workflow was used for daily online adaptation and treatment delivery. At each fraction, MRI sequences were performed, including T2nav, 3D VANE, and cineMRI BTFE sequences. T2nav was used for GTV delineation, and 3D VANE was used for delineating OAR and the whole spleen. The BTFE MRI provided a 2D cine-MRI on rapidly cycling single slices, in axial, coronal, and sagittal planes, which assess intrafraction motion and inform individualized ITV margins for each fraction. A 5-mm isotropic PTV margin expansion was subsequently applied. The IMRT plan was re-optimized online at each fraction (adaptive radiotherapy). Due to the close proximity of OARs, an isotoxic planning strategy was utilized, which prioritized OAR dose constraints over PTV coverage. As the target lesion was often unable to be visualized on the BTFE cine-MRI sequence, the whole spleen was used as a surrogate marker for target motion. A motion management (MM) structure was created by enlarging the spleen contour by the combined ITV and PTV margins.

Adaptive isotoxic planning resulted in the critical OAR being at or very near the constraint (especially D0.03cc) for every fraction. Improved target coverage was able to be delivered on fractions when greater separation between adjacent OARs and the targets was observed. The inter-fraction motion of the bowel resulted in variable distance to the target, which translated to >5-Gy (in five fractions) difference in both the PTV D95% and GTV D100% coverage for three of the patients. The range of delivered dose for each fraction to PTV D95% is shown in **Figure 2**.

**Figure 2:**
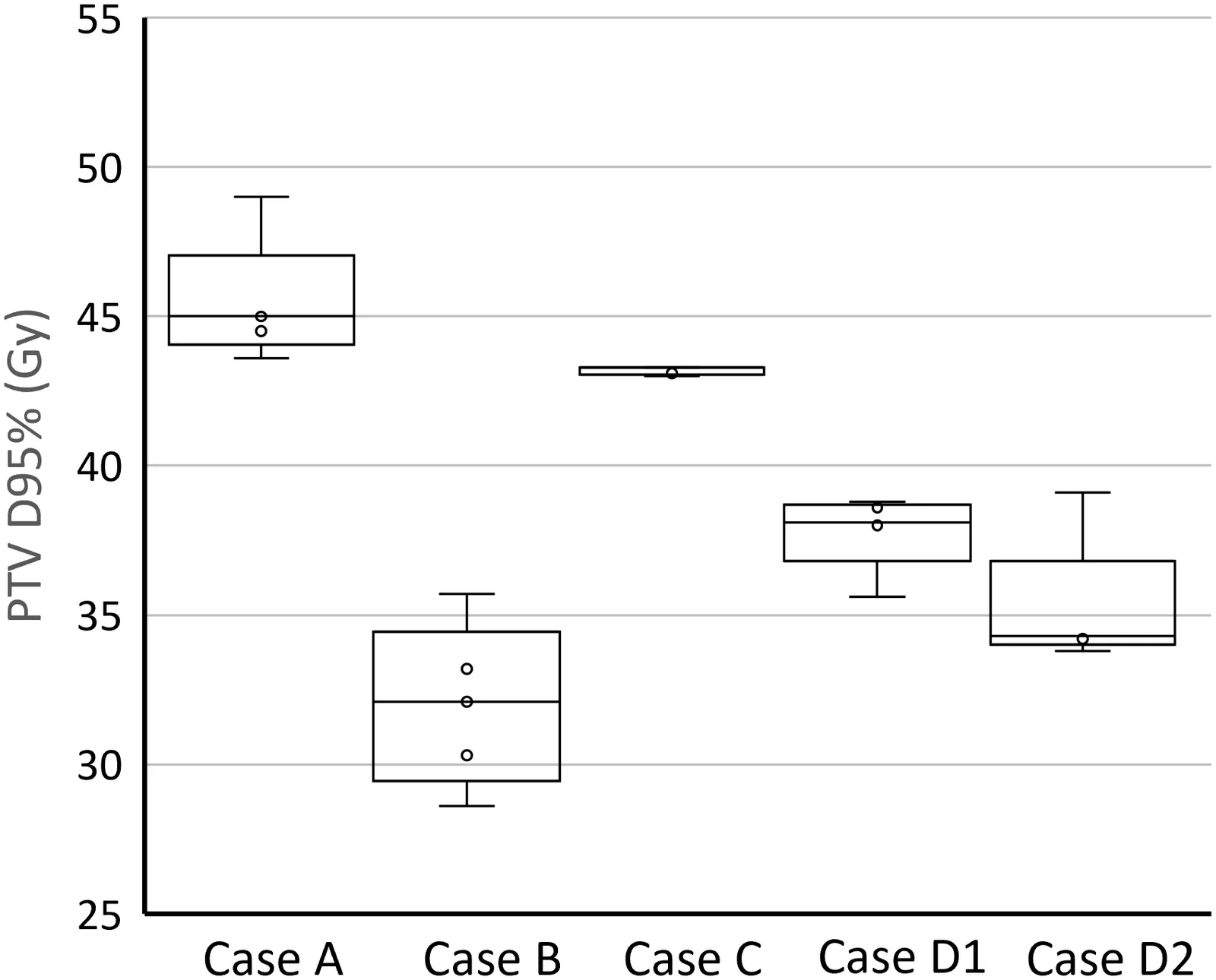
PTV D95% variability for each patient treatment (five fractions) and each fraction.

Cine-MRI was utilized during beam delivery, and if the spleen moved outside of the MM structure, the treatment would be manually interrupted until it returned inside the MM structure. OAR movement into the target volume can also be seen, and treatment is interrupted if it enters the MM. If the target/OAR remained out of position, then the remainder of the treatment MU/beams would be completed on another day.

Follow-up included blood tests, CT, or FDG-PET as determined by the treating oncologist. Toxicities were recorded as per CTCAE version 5 criteria (16).

The outcome and toxicity results are summarized in **Table 1**. Grade 2 lymphopenia was seen in one patient 3 months after his second spleen SBRT, and he recovered after 1 year. No grade 3+ acute or late toxicity was observed, including gastrointestinal or blood and lymphatic system disorders.

## Discussion and review of the literature

To our knowledge, this is the first report of SBRT for spleen metastasis delivered using MRgART, and we demonstrate good local control with minimal toxicity. Additionally, this study represents the first documented use of splenic SBRT for metastatic melanoma and colorectal adenocarcinoma as well as the first reported case of splenic SBRT re-irradiation.

There are only a few published case reports of splenic SBRT to date, as summarized in **Table 2**. Previous cases typically utilized either robotic or conventional LINAC and similarly reported excellent local control with minimal toxicity, including hematological adverse effects. One specific case reported initial pseudoprogression at 1 month post-SBRT, followed by a complete metabolic response on subsequent imaging. In comparison to our cohort, the previously published splenic oligometastases were predominantly located in the central, posterior, or lateral regions of the spleen, which are away from adjacent bowel structures.

**Table 2:** Comparative summary of case reports.

Author	Primary	Age	Dose	Technique	Outcome	Toxicity
Chen 2016 (12)	Thymic carcinoma, metachronous, 45 mm	38	30Gy/5#	Fiducial+ robotic LINAC	Partial response at 1 month	
Juloori 2019 (10)	NSCLC, metachronous, 13 mm	61	50 Gy/5# daily	Expiratory breath hold; conventional LINAC	Local control >52 months	Parvovirus infection
Gupta 2025 (11)	Gastric adenocarcinoma Metachronous, 15 mm	50	50 Gy/5# alternate daily	DIBH; conventional LINAC	Stable disease at 7 months	G1 fatigue, nausea, vomiting, diarrhea No toxicity at 14 months
Hughes 2019 (8)	Ovarian epithelial	85	50 Gy/10#	Abdominal compression, conventional LINAC	Partial response at 12 months	G1 fatigue, G1 anaemia; reduced spleen volume
Kim 2020 (9)	NSCLC, oligoprogression, 36 mm	80	50 Gy/5#	Fiducial marker, ITV conventional LINAC	Complete response 10 months	Pseudo-progression at 1 month

Alternative radiotherapy prescriptions for gastrointestinal targets include fractionated radiation and chemoradiation. Doses for both are limited by bowel constraints, and we consider SBRT generally able to deliver a higher biological effective dose and local control and is logistically easier for the patient.

Particular challenges for spleen SBRT include spleen inter- and intrafraction motion and close proximity to luminal organs (stomach, small bowel, and large bowel), where exceeding dose constraints increase the risk of severe complications such as ulceration, perforation, bleeding, and/or stricture. The spleen is an intraperitoneal organ, with ligaments to the stomach, kidney, and diaphragm, and as such its position moves depending on breathing, posture, gastric filling, and motion of other organs. Two prior case reports utilized a fiducial marker, the insertion of which has a significant bleeding risk when performed percutaneously under US or CT guidance, and the alternative of endovascular insertion is more invasive (3). Fiducials allow visualization on CBCT and KV tracking; however, they cannot monitor OAR movement. An MRI-based study found that, excluding respiration, OAR movement of the stomach and small and large intestine ranges from 5 to 20 mm over 16 min, with a median of 8–14 mm (17). MR-guided radiotherapy does not need fiducial placement and allows daily visualization of the target, whole spleen, and OAR. Real-time cine-MRI allows intrafraction tracking of the spleen and adjacent critical OARs, allowing safe delivery of SBRT.

Online adaptation allows treatment plan re-optimization based on daily anatomical variations. This is particularly important for three of our cases where the target volume abutted the adjacent luminal OARs such as stomach and/or bowel. Employing an isotoxic planning strategy allowed the delivery of a high dose to the GTV by selectively reducing PTV coverage when necessary.

With daily anatomical variation, we were able to deliver a higher PTV dose when increased separation between the target and OARs was observed. This is evident for patient B, who showed a 20% variation (7.1 Gy in five fractions) in daily PTV D95%, ranging from 28.6 to 35.7 Gy. These doses correspond to a biological equivalent dose (BED_10_) of 45 vs. 61 Gy. Without adaptation, a stomach planning organ at risk volume (PRV) constraint (often 3 mm) would further compromise target coverage. Furthermore, should anatomical variations have exceeded the PRV, the stomach could have received doses exceeding the safety constraints, increasing the risk toxicity.

The limitations of our series include the absence of histopathological confirmation, the lack of long-term follow-up for toxicity, the inherent heterogeneity of the patient cohort, and their diverse prior oncological treatments. All patients were discussed at MDT, and based on the patients’ imaging findings, oncological history, and the potential risk of splenic hemorrhage, splenic biopsy was not recommended.

Regarding motion management, alternatives to the ITV/abdominal compression technique such as breath holding or respiratory gating may offer increased precision and allow for reduced motion margins (18). However, the disadvantages of these techniques include a lower duty cycle, potentially increasing a standard 10-min beam-on time to 20–30 min, and the noise of the MRI machine can make verbal coaching and compliance challenging. Comprehensive motion management (respiratory gating) has recently been developed for the Elekta Unity but was not available to be utilized in this case series (19).

There is uncertainty regarding an optimal OAR constraint for the spleen for both fractionated radiotherapy and SBRT. Retrospective studies of patients treated with chemoradiotherapy for gastric, esophageal, and pancreatic cancers suggest a relationship between V5, V15, Dmax, and Dmean and lymphopenia, with a possible threshold of Dmean 9–18 Gy for grade 3 lymphopenia (3, 4). The Royal College of Radiologists guidelines recommend considering long-term hyposplenism prophylaxis if Dmean is >10 Gy (20). For SBRT, protocols generally do not specify a spleen OAR constraint (21). We saw no severe hematologic toxicity with 10–22 Gy mean spleen dose; however, we may have seen less toxicity as our patients were not on concurrent chemotherapy. Longer-term assessment and follow-up regarding hyposplenism and infectious sequelae are therefore essential. Asplenic patients are at risk of encapsulated bacteria, malaria, and overwhelming sepsis, and recommendations are for pneumococcus, HIB, and meningitis vaccinations and daily antibiotic prophylaxis for 2 years.

Future studies, with more patients, should compare spleen SBRT to the standard treatment of surgical splenectomy. They could include measures of spleen function to more accurately assess hyposplenism. Functional spleen assessment may include pitted erythrocyte count, 99m-technetium-labeled heat-denatured red cell scans, or 99m-technetium-labeled sulfur colloid scans (22). Other future directions also include evaluation of MRgART patient-reported outcomes and evaluation of the ideal dosimetric constraints for spleen irradiation.

## Conclusion

This case series demonstrates the technical and clinical feasibility of MRgRT splenic SBRT. Our experience suggests that MRgRT provides a non-invasive treatment option with encouraging disease control and an acceptable toxicity profile. However, this approach should be carefully balanced against the risk of hematological toxicity, and the implementation of infection prophylaxis should be strongly considered in the clinical management of these patients.

## Data Availability

The raw data supporting the conclusions of this article will be made available by the authors, without undue reservation.
